# Emergency treatment of pelvic ring injuries: state of the art

**DOI:** 10.1007/s00402-024-05447-7

**Published:** 2024-07-06

**Authors:** Jan Erik Madsen, Gunnar Birkeland Flugsrud, Niels Hammer, Paul Puchwein

**Affiliations:** 1 Division of Orthopaedic Surgery, Oslo University Hospital, Kirkeveien 166, 0450 Oslo, Norway; 2 Institute of Clinical Medicine, University of Oslo, Klaus Torgårds Vei 3, 0372 Oslo, Norway; 3 Division of Macroscopic and Clinical Anatomy Gottfried Schatz Research Center, Medical University of Graz, Graz, Austria; 4 Department of Orthopaedic and Trauma Surgery, University of Leipzig, Leipzig, Germany; 5 Division of Medical Technology, Fraunhofer Institute for Machine Tools and Forming Technology (Fraunhofer IWU), Dresden, Germany; 6 Department of Orthopedics and Trauma Surgery, Medical University of Graz, Graz, Austria

**Keywords:** Pelvic fracture, Hemorrhage, Initial treatment, Damage control resuscutation

## Abstract

High energy pelvic injuries sustain significant mortality rates, due to acute exsanguination and severe associated injuries. Managing the hemodynamically unstable trauma patient with a bleeding pelvic fracture still forms a major challenge in acute trauma care. Various approaches have been applied through the last decades. At present the concept of Damage Control Resuscitation (DCR) is universally accepted and applied in major trauma centers internationally. DCR combines hemostatic blood transfusions to restore blood volume and physiologic stability, reduced crystalloid fluid administration, permissive hypotension, and immediate hemorrhage control by operative or angiographic means. Different detailed algorithms and orders of hemostatic procedures exist, without clear consensus or guidelines, depending on local traditions and institutional setups. Fracture reduction and immediate stabilization with a binder constitute the basis for angiography and embolization (AE) or pelvic packing (PP) in the hemodynamically unstable patient. AE is time consuming and may not be available 24/7, whereas PP offers a quick and technically easy procedure well suited for the patient in extremis. Resuscitative endovascular balloon occlusion of the aorta (REBOA) has also been described as a valuable adjunct in hemostatic non-responders, but merely constitute a bridge to surgical or angiographic hemostasis and its definitive role in DCR is not yet clearly established. A swift algorithmic approach to the hemodynamically unstable pelvic injury patient is required to achieve optimum results. The present paper summarizes the available literature on the acute management of the bleeding pelvic trauma patient, with emphasis on initial assessment and damage control resuscitation including surgical and angiographic hemostatic procedures. Furthermore, initial treatment of open fractures and associated injuries to the nervous and genitourinary system is outlined.

## Introduction

Trauma patients with bleeding pelvic injuries constitute a significant challenge in trauma care. Historically they carry a high risk of severe complications and death in up to 60% of cases [29, 36, 37, 125, 172, 177, 178]. Predictors of mortality have been extensively studied, and recalcitrant hemorrhage during the first 24 h constitute the main cause of mortality [3, 78, 124, 134]. In addition to ongoing hemorrhage and pelvic ring instability, high age, Injury Severity Score (ISS), severity of soft tissue injuries including wound size and rectal injury in open fractures, as well as head Abbreviated Injury Scale (AIS) and admission base deficit, constitute independent mortality predictors. Appropriate recognition of the extent of injury, as well as a multidisciplinary approach to initial resuscitation, hemostatic measures and proper management of other urgent injuries, are key to survival for these patients.

Significant variations in trauma system layout and initial treatment algorithms persist, depending on local geography and traditions, hospital designs, as well as available resources in the acute setting [16, 19, 65, 114, 120]. Acute hemostatic damage control measures like fracture stabilization, angiography with embolization (AE) and preperitoneal pelvic packing (PP) were introduced decades ago and have been extensively documented. The introduction of the Damage Control Resuscitation (DCR) concept about 20 years ago constituted a main paradigm shift, by incorporating modern resuscitation algorithms based on hypotensive fluid resuscitation and hemostatic transfusions with the hemostatic damage control procedures in a structured concept [12, 54, 64]. The DCR concept has shown a potential to lower mortality and decrease the need for acute hemostatic interventions like AE and PP [64] and was summarized in treatment guidelines formulated by the World Society of Emergency Surgery in 2017 [33].

Resuscitative endovascular balloon occlusion of the aorta (REBOA) was firstly used in exsanguinating warfare injuries but has recently also been introduced as a damage control measure in cases of exsanguinating hemorrhage in civilian trauma systems [175, 130]. REBOA mainly acts as a bridge to other treatment modalities, and its role in the acute treatment of the exsanguinating pelvic trauma patient is not yet clearly established.

This present manuscript aims to summarize recent advances in DCR of the unstable pelvic fracture patient, and outline recommendations for the initial treatment of the severely injured pelvic fracture patient.

### Initial assessment of the pelvic fracture patient

#### Clinical assessment

Due to the high trauma energy involved, severe pelvic trauma rarely presents as single injuries. Up to 80% associated injuries to CNS, thorax and/or abdomen has been reported [37, 44, 173], emphasizing the importance of multidisciplinary teamwork and effective initial polytrauma protocols. Primary trauma team evaluation according to the Advanced Trauma Life Support (ATLS) guidelines has been widely adopted internationally and provides an effective and versatile system for identification and initial treatment of life-threatening injuries [59]. The ATLS guidelines have also defined the term hemodynamic instability, as systolic blood pressure < 90 mmHg, heart rate > 120, altered consciousness and/or shortness of breath, and classified hypovolemic shock into four classes based on clinical and physiological parameters, to serve as a resuscitation guide.

The severely injured pelvis can accommodate 3000 mls of blood and may bleed up to 1000 ml/hour [99], and bleeding is the main cause of mortality after trauma [3, 124, 134]. Thus, hypovolemia must be carefully evaluated, and a hemorrhagic shock diagnosed, graded, and treated promptly. Tachycardia and cool peripheries may be early indicators of significant blood loss, whereas relying on initial blood pressure can be misleading, as up to 30% of the blood volume can be lost before hypotension occurs. It is of utmost importance to identify bleeding sources early, and the diagnostic workup must be standardized and streamlined for this purpose.

The physical examination in suspected severe pelvic injury includes deformities, thorough inspection of soft tissues, clinical evidence of genitourinary injury, and exclusion of perineal injury/open pelvic fracture by rectovaginal examination. If a urethral injury is suspected, a careful try passing a urethral catheter should still be performed. If unsuccessful, a suprapubic catheter is indicated, together with further urogenital diagnostics [15, 109]. Leg length discrepancies and rotational deformities may indicate severely displaced pelvic or acetabular fractures, and in the awake patient palpation for local bony tenderness may be of value. A logroll is to be performed with utmost care, and not before a pelvic fracture is properly stabilized, to prevent dislodgments of established clots [59, 132].

#### Imaging

The initial diagnostic adjuncts in the ATLS protocols focus on identifying life threatening injuries and acute bleeding sources. The Extended Focused Assessment with Sonography for Trauma (eFAST) is now widely used to quickly detect significant blood collections in the peritoneal and thoracic cavities, and it can also be helpful in determining appropriate zone placement for REBOA (I vs. III) [32]. The method is to some extent examiner-dependent but is quick and generally easily available in the ER. Its diagnostic accuracy has been extensively studied, and high specificities (90–100%) suggest that it is an excellent rule-in tool for thoracic and abdominal bleedings, whereas lower sensitivities (70–80%) makes it less suited for ruling out severe bleedings [121].

Displaced pelvic fractures can easily be diagnosed with a plain antero-posterior (AP) pelvic radiograph in the ER. A displaced pelvic fracture indicates a severe injury with potential hemorrhage and paves the way for immediate damage control procedures like fracture reduction and stabilization without further diagnostic delay. Undisplaced posterior ring fractures may be missed on the initial pelvic radiograph, but the radiograph is still of significant value for the trauma team when evaluating the extent and spectrum of injuries.

For exact and complete diagnosis and classification of a pelvic disruption, CT is mandatory. Whole-body CT (WBCT) protocols have gradually overtaken previous select protocols, and provide unenhanced head and cervical exams, and contrast-enhanced chest, abdomen and pelvis CTs with acceptably low radiation loads [34]. Still, the persistently unresponsive hypotensive patient should undergo immediate damage control procedures before performing a CT scan due to the risk of circulatory collapse.

### Damage control resuscitation

Acute hemorrhage accounts for up to 40% of trauma deaths and may be the leading cause of preventable deaths in trauma [154]. Recent research into military and civilian trauma populations has focused on ways to improve survival in the case of severe hemorrhage. The term “damage control” in traumatology was adopted from the Navy definition being “the capacity of a ship to absorb damage and maintain mission integrity” [47] and coined in surgery by Rotondo et al. in 1993 [147]. The damage control (DC) principle in surgery was based on immediate arrest of major bleedings, mainly by sponge-based packing and abbreviating operative interventions; everything to avoid the potentially lethal triad of hypothermia, coagulopathy and acidosis [147]. Once damage control surgery was established, the term was expanded into other disciplines like vascular, thoracic, military and orthopedic injuries [155].

DCR has gradually developed from the initial concept of damage control surgery and was firstly delineated by Hess et al. in 2003 [82]. It currently includes hemostatic blood transfusions to restore blood volume and physiologic stability, reduced crystalloid fluid administration, permissive hypotension, and immediate hemorrhage control by operative or angiographic means.

The early coagulopathy in trauma is a well-recognized and feared entity, and the understanding of its pathophysiologic complexity has gradually increased. Historically, the trauma induced coagulopathy was considered a consequence of hemodilution, hypothermia and resuscitation, whereas later studies have emphasized a more complex understanding where tissue trauma, hypothermia, shock, acidosis and inflammation are all considered to play key roles [81]. This understanding indeed supports the modern use of massive transfusion protocols (MTPs), aiming to deliver fresh whole blood approximations of RBCs, fresh frozen plasma and platelets in a 1:1:1 ratio [21, 82]. The MTPs have reduced mortality in multiply injured patients [21, 46, 64, 83, 84], and significantly reduced the need for surgical and angiographic hemostatic emergency procedures in patients with bleeding pelvic fractures [64]. Despite improved resuscitation strategies, in Gaski’s paper 37% of the severe pelvic fractures with high transfusion needs were still subjected to PP as a damage control/salvage procedure, indicating that surgical and angiographic hemostatic procedures remain important cornerstones in the DCR algorithms.

Today’s DCR incorporates the concepts of hemostatic transfusions, permissive hypotension, and protection from hypothermia, with acute hemostatic surgical and/or angiographic procedures like fracture reduction and stabilization, PP, AE and REBOA. Despite widespread adoption of the DCR concept, evidence-based guidelines are not universally agreed upon, and algorithms differ significantly according to geography, trauma load, and resource availabilities.

#### Tranexamic acid

The lysine analogue tranexamic acid (TXA) is an antifibrinolytic drug, extensively studied in trauma. It prevents clot breakdown and reduces blood loss in exsanguinating trauma [107, 142]. The CRASH-2 randomized control trial included over 20,000 trauma patients with or at risk of significant bleeding and showed that mortality rates were reduced with about one third with TXA administration within 3 h of trauma: from 7.7 to 5.3% given within 1 h, and from 6.1 to 4.8% if given between 1 and 3 h. Delayed TXA-administration (after 3 h) increased mortality due to hemorrhage from 3.1% to 4.4%. The authors concluded that TXA should be given as early as possible to bleeding trauma patients, whereas late administration is less effective and potentially harmful [142]. Current evidence thus supports the routine use of TXA in bleeding pelvic trauma patients, and TXA should be included in present transfusion protocols [107, 141].

### Hemostatic damage control procedures

DCR includes damage control hemostatic procedures in the hemodynamically unstable patient with a bleeding pelvic injury. The sequence of procedure application may vary significantly between trauma centers, according to trauma load, availability of resources, institutional setup with/without hybrid ERs/ORs and local traditions. In the following, we line up an algorithm suited for a trauma center with a hybrid ER/OR, incorporating all operative and angiographic facilities without the need for inline transport of the patient.

#### Fracture reduction and stabilization using the pelvic binder

Bleeding from the injured pelvis occur from displaced fracture surfaces, veins, arteries and soft tissues, with veins and fracture surfaces representing the main bleeding sources [87, 113]. As the pelvic volume increases with 10–20% by 5 cm opening up of the pelvic ring [148], closing up and stabilizing the pelvic ring represent a first line measure to halt low pressure hemorrhage, by limiting space and facilitate early tamponade. A pelvic binder is the preferred mean for stabilizing a pelvic fracture in the acute setting; it is fast, cheap, and easily applicable. Correctly placed over the trochanters, the binder reduces pelvic volume and improves hemorrhage control [11, 22, 38, 43, 96]. A binder is frequently applied in the pre-hospital setting, often without taking the importance of fracture reduction into account. If a significant fracture displacement is present on initial AP pelvic radiograph in the ER, with ongoing hemodynamic instability, it is therefore recommended to re-apply the binder following proper fracture reduction. This is performed by manual traction and/or rotation of one or both lower legs, according to the fracture displacement pattern, before re-applying the binder. The pelvic binder could be a simple folded sheet or a commercially available one, both documented to provide sufficient stability provided proper placement over the trochanters [11, 135]. Binder compression over the trochanters place significant compressive forces to the posterior pelvic ring and leaves the abdomen free for damage control procedures like explorative laparotomy and pelvic packing.

Some fracture patterns, like acetabular fractures and other lateral compression type fractures, may not comply with the binder concept as a primary stabilizer, as this may further distort the pelvic ring anatomy. The fear that this might potentially worsen bleedings is not, however, supported in the literature [11]. In such cases an initially applied binder can be replaced with an external fixator or a C-clamp as soon as this is available in the hemodynamically unstable patient. If hemodynamics stabilizes, lateral compression type fractures can in many cases be left without further external support after binder removal, while awaiting final fracture fixation.

A tightly placed binder may exert soft tissue pressures exceeding the limit for adequate skin circulation [136] and may result in severe soft tissue damage if left more than 24 h [9, 11, 19, 125]. We therefore recommend releasing the binder in the ICU the morning after trauma, provided regained hemodynamic stability. If fracture displacement and/or instability require further fracture stabilization the binder can be converted to final ORIF, or an external fixator according to the patient’s trauma load and physiologic condition.

#### External fixators

Anterior external frames and C-clamps have both been variably used to stabilize an unstable pelvic fractur, but their application is generally more time consuming compared to a binder and require the expertise of a trained orthopedic or trauma surgeon. Anterior frames were historically applied with multiple Schanz pins placed in the iliac crest [139, 160]. These constructs tended to interfere with abdominal procedures and were prone to early loosening due to inferior screw purchases and misplacements. Therefore, the supraacetabular route for Schanz pin placement should be preferred when applying anterior frames [133]. This frame can easily be applied in a hybrid ER or OR with or without fluoroscopy depending on the surgeon’s level of expertise and enables a simpler frame construct that will not interfere with abdominal or pelvic hemostatic emergency procedures. The supraacetabular pin configuration also creates better compressive forces to the posterior pelvic ring compared to iliac crest frames [164].

C-clamps were introduced more than three decades ago [62] for stabilizing and compressing the posterior pelvic ring. Experimental data showed that average forces of up to 342 N could be applied to the SI-joints, and fracture reduction and hemodynamics improved provided correct application [62]. Mechanically, a posteriorly placed device is superior to an anterior one, and the C-clamp provides good access to the abdomen and groins for acute hemostatic procedures. In experienced hands the C-clamp can be fast and easy to apply, but fluoroscopic guidance is advised to avoid misplacement of pins and over-compression of sacral fractures. Complications like pin site infections and neurovascular injuries due to pin misplacement into the true pelvis, or overcompression of the clamp has been reported [61, 158]. Together with the fact that the need for hemostatic emergency procedures have grossly diminished due to improved resuscitation protocols [64], this has led to a reduction in the use of the C-clamp in many trauma centers [10, 158].

**Fig. 1:**
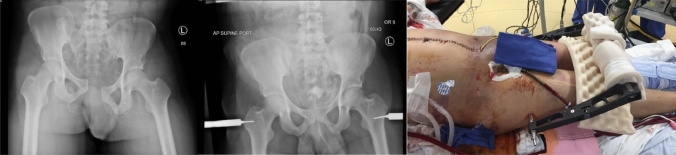
Application of the C-clamp as a T-clamp without fluoroscopy control during damage control resuscitation ensures rapid fracture stabilization and excellent access to abdomen and groins for operative interventions. Printed with permission from Sepehri et al. [159]

An efficacious modification of the C-clamp application was published by Archdeacon et al. in 2006 [7], and later studies confirmed this modification to mount the C-clamp directly onto the major trochanters and thereby exerting its compressive forces to the posterior pelvic ring through the hip joints [8]. This technique, termed the T-clamp, is fast and easy to apply without fluoroscopic control, and the frame can also in most cases be left in place until final fracture fixation of the posterior pelvic ring can be performed, without an elevated risk of infectious complications [159] (Fig. 1).

#### Angiography and embolization

It is generally assumed that the main sources of bleeding in pelvic trauma is from the low-pressure systems [13, 87]. Even so, significant arterial bleedings have been documented in 10 to 20% of cases, and up to 60% of cases if the patient is hemodynamically unstable [50, 60, 77, 113, 114, 171]. AE has been in practice for pelvic trauma since the early 1970’s [108] and has proven safe in selected patients, and efficient in controlling pelvic arterial hemorrhage [19, 60, 78, 101, 103, 126, 144, 171]. Even so, the mortality rates may be high in this trauma population, especially in the older clinical series [103, 171, 176].

Pelvic trauma patients remaining hemodynamically unstable during massive transfusions and after appropriate fracture reduction and stabilization are potential candidates for pelvic AE. Specific indications are still being debated [60, 171], and no standard protocol based on consensus is established. Some authors have sought to predict the need for AE based upon fracture classification and/or vital signs, and even though the most unstable fracture patterns are at great risk for arterial bleedings, it has been difficult to predict which patient will benefit the most from AE [19, 50, 70, 90, 122, 144, 163]. Several authors have investigated the use of contrast enhanced CT to predict the need for AE, and indeed an arterial blush and a pelvic hematoma seem to predict the need for AE with high sensitivity (60–90%) and specificity (92–100%) [14, 72, 114, 129, 169]. In our opinion, contrast enhanced CT is helpful in determining the need for AE, but only in the context of persisting hemodynamic instability despite ongoing adequate resuscitation.

Compared to pelvic packing, AE is less invasive but requires specialized service 24/7 and is therefore not available acutely in all trauma centers. Also, the time from arrival to completed AE is up to 2–3 h in several publications [101, 171]. If the patient needs transferal from the ER to a distant angiography lab, AE is difficult to see as a primary hemostatic measure for the critically ill trauma patients in hemorrhagic shock. Hybrid ERs/ORs have, however, significantly shortened the AE procedure time and it is at present our preferred damage control option in patients remaining in hypovolemic shock during adequate resuscitation.

The most frequent arterial bleeders are branches from the internal iliac artery [156, 171, 176], and AE should be performed as selectively as possible to avoid severe gluteal soft tissue complications [103, 110, 180]. Previous empiric bilateral internal iliac artery embolization had an increased risk for serious complications like pelvic ischemia, gluteal muscle and other soft tissue necroses [48, 166, 182] and can no longer be advocated.

Of note is also the fact that high energy acetabular fractures are hampered with a significant arterial hemorrhage potential; In Tötterman’s study comprising 31 patients undergoing AE for exsanguinating pelvic injuries, 4 of 31 patients had isolated acetabular fractures and bleedings from internal iliac branches [171].

#### Pelvic packing

Preperitoneal PP was introduced as a salvage procedure to control massive pelvic fracture related hemorrhages [52, 134, 140] and has proven efficient also in patients with multiple bleeding sources, both intra- and retroperitoneal [64, 67, 100, 161, 173]. The procedure can be performed in less than 30 min in the ER and also in conjunction with an exploratory laparotomy if indicated. Some centers have advocated PP as the preferred initial hemostatic procedure after fracture reduction and stabilization [100, 125], as it is quickly accomplished and addresses both venous and fracture surface bleedings [24, 101, 168]. Several studies have compared AE with PP in this context and concluded that treatment algorithms including PP could reduce mortality and transfusion requirements compared to AE [57, 100, 101, 125], even though obvious selection biases preclude conclusive evidence. Thus, there is at present no clear evidence to support the superiority of either PP or AE for the other [119, 167]; and the two methods should be considered complementary rather than competitive tools. Tötterman et al. reported on 18 pelvic fracture patients in extremis undergoing PP, and subsequent AE. 80% (15/18) of the patients had ongoing arterial bleedings after PP and profited from additional embolization. Thus, PP and AE represent parts of a multi-interventional resuscitation protocol, and we have continued utilizing this combined approach. For similar reasons, survival rates after pelvic packing will inevitably vary depending on the indication for its use [100]. Ron et al. reported 100% survival using PP as a stand-alone treatment [143], whereas Tötterman et al. reported 30-day survival of 72% in a trauma population with a mean ISS of 48 [173] and Papakostidis` review estimated an overall mortality rate of 28% [127].

PP has a potential for significant complications. Papakostidis estimated a 35% infection rate in his review [127] and15% infections were reported by Burlew in a series of 75 patients subjected to PP, with 47% infections after re-packing due to residual hemorrhage after removal of packs [25]. The infectious complications were probably mostly related to open fractures with severe soft tissue compromise and associated bowel/bladder injuries, and also in Tötterman’s report 5 of 6 infections occurred in open fractures [173]. Because of this, pelvic packings should be removed as early as possible, we prefer before 24 h, typically first day after PP, and we also try to avoid ORIF of the anterior pelvic ring in high-risk cases. Deep venous thrombosis (DVT) is also reported after PP, supporting early DVT screening in these patients [25, 79].

**Fig. 2:**
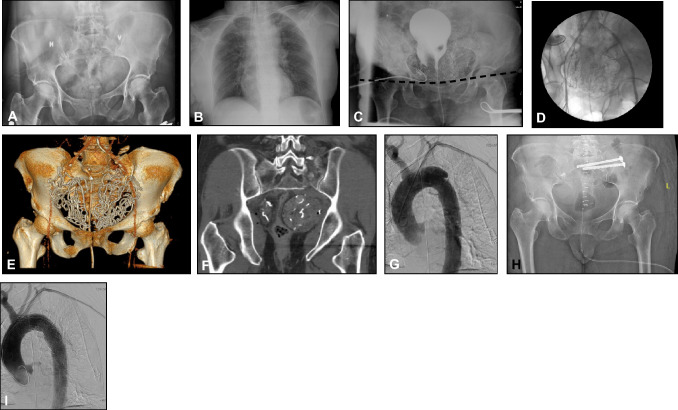
Case of a 58-year-old female jumping off pedestrian bridge arriving ER 17 min after trauma in class IV hypovolemic shock. **A** Vertical shear type pelvic fracture. **B** Chest x-ray with slightly widened mediastinum. **C** Pelvic fracture reduced and stabilized with pelvic binder, and pelvic packing completed 32 min after arrival. Dotted line represents upper border of the binder. **D** Persisting hemodynamic instability indicated angiography and embolization of right sided bleeders. **E** and **F** CT illustrating correctly placed pelvic packs. **G** The contained aortic rupture. **H** and **I** Unpacking, aortic stenting and pelvic fracture fixation performed on day 1

Patients remaining in extremis during aggressive resuscitation, including massive transfusions, fracture reduction and stabilization, may not survive the procedure time for AE and should be subjected to PP in the ER without further delay (Fig. 2).

#### Bilateral internal iliac artery ligation

In conjunction with PP or laparotomy, internal iliac artery (IIA) ligation has been advocated in austere locations such as rural hospitals of developing countries and military combat zones, without AE services available. The surgical procedure involves careful dissection of the IIA and vein close to the bifurcation after PP has been performed, which is time consuming and technically demanding. Procedure times over 1 h is reported and required removal of the pelvic packs [31]. Iatrogenic complications such as external iliac artery occlusion, internal and external iliac vein damage with consequent lower extremity amputations is reported, and mortality rates remain high [31, 48]. Chernobylsky compared 112 pelvic fracture patients undergoing AE with 51 patients undergoing IIA silastic loop ligation. The IIA ligation group had higher infection rates, reoperation rates and transfusion burden, and mortality rate was 57% compared with 23% after AE [30].

In modern trauma centers we do not consider IIA ligation to be a necessary part of the damage control resuscitation algorithms, but in unfavorable scenarios it may constitute a life-saving alternative to AE after PP.

#### Resuscative endovascular balloon occlusion of the aorta (REBOA)

REBOA was first described used in major exsanguinating injuries during the Korean war in the 1950s [85]. It has gradually been developed for use also in civilian trauma centers for non-compressible hemorrhages in the thorax, abdomen and pelvis [75, 106] and in 2014 the Joint Theater Trauma System released US guidelines implementing REBOA in the algorithm for treating profound shock [175].

The clinical use of REBOA at present differ according to geography and institutional algorithm designs, and the reported clinical results are conflicting. Some authors have reported significant incidences of severe complications, like lower limb ischemia and amputation [152], and improved survival rates have been difficult to prove [123]. A US multicenter study compared resuscitative thoracotomy with REBOA and showed equal times to aortic occlusion (7 min), and an overall survival rate of 24 of 114 patients, with few complications related to the REBOA [49]. However, a recent US case control study of 420 trauma patients showed that REBOA-placement resulted in severe complications and increased mortality [93], and in the UK REBOA trial mortality from early pelvic bleeding was doubled in the REBOA + standard care group compared with the standard care alone group (32 vs. 17%) [88].

Aortic occlusion creates total lower body ischemia, and occlusion times are critical; they must be kept within safe limits, not well defined in a trauma population. Partial balloon occlusion seems to preserve distal blood flow to some extent and can therefore probably extend the occlusion time and its clinical implementation seems to increase [55, 56, 111, 150].

In severe pelvic trauma with exsanguinating hemorrhage, where available, the REBOA is recommended to be placed immediately over the aortic bifurcation, in zone III. Increased precision of balloon placement can be achieved using fluoroscopy or eFAST [28, 49, 74]. Introduction of the balloon through 6 or 7 French introducers can often be performed percutaneously and is hampered with less introducer site complications compared to first generation 12F introducers, frequently requiring open vascular approaches and repairs. Procedure time until balloon inflation, however, may not differ significantly between percutaneous and open technique [71].

One needs to consider that REBOA may worsen the outcome for certain injuries and may be considered contraindicated in i.e. bleeding penetrating neck and chest trauma, where a resuscitative thoracotomy constitutes the first line of treatment. Furthermore, when a contained aortic injury is suspected, REBOA must be used with caution (Fig. 2). Also, traumatic brain injury was previously a source for concern, but is no longer considered a contraindication for REBOA [23].

The ultimate role for REBOA in the setting of exsanguinating pelvic trauma is not yet clearly established. All providers should be aware that the technique of REBOA is a hemorrhage control adjunct only and must be considered a bridge to other hemostatic damage control procedures. Since it is critical to minimize the duration of balloon inflation, the patient should immediately be taken to an OR or angiography suite for pelvic packing or AE, according to institutional capabilities [40].

#### Acute internal fixation

Gardner and Routt described a case where they successfully treated a trauma patient with a dislocated SI joint in hemorrhagic shock due to pelvic bleeding, using acute closed reduction and SI screw fixation [63]. They termed this the “antishock iliosacral screw” (ASISS) and claimed it to be an excellent resuscitative adjunct for the patient with displaced posterior pelvic ring injuries amenable to compressive reduction. This ASISS technique has later been promoted by renowned and experienced pelvic trauma surgeons, but very little has been published on the issue. In our experience, the technique is not suited for the pelvic trauma patient in extremis, that is in persisting hemorrhagic shock despite fracture reduction, stabilization, and ongoing adequate transfusions. Placing SI screws requires careful planning of screw trajectories with a completed CT scan, and the patient needs to be transferred to proper OR facilities, with a highly experienced pelvic surgeon attending. Also, despite proper fluoroscopic conditions, closed reduction may be difficult to evaluate [97], especially in sacral fractures, and the safe sacroiliac (SI) screw corridors can easily be compromised by lack of fracture reduction as well as unrecognized sacral dysmorphisms.

In the original paper it was emphasized that this procedure is extremely demanding in a stressful acute situation. Additionally, the initial resuscitation and preparations for an acute internal fixation will easily consume 2–3 h, which is too long for a patient in extremis. The term “antishock iliosacral screw” therefore seems misleading, and the acute fracture fixation should more likely be termed “early internal fixation”. If early SI screw fixation is to be used, we suggest using it for SI joint dislocations, and avoiding sacral fractures due to difficulty in evaluating fracture reduction, with potentially compromised safe screw corridors.

### Associated injuries

#### Injuries to the genitourinary system

Injury to the genitourinary (GU) system is most frequent in men, often involving the posterior urethra or the extraperitoneal bladder; only two of twenty GU injuries concurrent with pelvic fractures (10%) occurred in women in Jensen’s series [89]. Also, among 25 females with pelvic fracture and urethra/bladder injury, only 14 (56%) were diagnosed in the ER [20], showing that a high level of suspicion is needed as female urethral injuries should preferably be repaired as soon as possible [98].

In men, clinical signs include blood at the meatus and a high-riding prostate, whereas perineal hematoma may be a later sign. If suspecting urethral damage, one gentle attempt at urethral catheterization is warranted [15, 116]. If this fails the catheter is removed, and a suprapubic catheter introduced under ultrasonographic guidance. When undergoing CT scan, delayed images should be obtained during the excretory phase. When needed, a retrograde urethrogram may be added, installing up to 30 ml of contrast through a retrograde catheter gently inflated a few centimeters beyond the meatus. If a suprapubic catheter is placed, an antegrade cystourethrography may be performed installing up to 300 ml contrast through the catheter [98].

Classification of urinary injuries focus mainly on the anatomic localization (anterior or posterior to the urogenital diaphragm), and on the degree of injury (stretch, partial rupture, complete tear) [69, 109].

With extraperitoneal bladder ruptures The American Urological Association advices early surgical repair and catheter drainage [116]. Saiz found that among 68 patients with pelvic fracture and a concomitant extraperitoneal bladder rupture, four of thirteen patients neither undergoing anterior internal fracture fixation nor bladder repair developed deep infection (31%), whereas only one deep infection occurred among the 53 patients who had bladder repair and anterior internal fixation (41 with plates) [153]. Bladder ruptures rarely leads to late sequelae [89].

Controversy remains concerning posterior urethral injury in males with pelvic fracture. Initial treatment may be primary realignment (PR) [51] performed together with anterior internal fixation, or primary suprapubic catheter and delayed urethroplasty (SPCDU). Patients with this injury pattern have high rates of late strictures, erectile dysfunction and incontinence [15]. While urologists consider SPCDU to have superior long-term results, and orthopaedic surgeons consider suprapubic catheter to increase infection rate when they perform anterior pelvic internal fixation [91], there is a paucity of literature allowing these factors to be evaluated together [15, 102].

Our practice when doing anterior internal fixation is to repair an extraperitoneal bladder rupture, and to perform primary realignment for the posterior urethral injury, aiming to reduce deep infections and the need for later urethroplasty.

#### Nerve injury

The pelvic area includes the part of cauda equinae containing the L5 and the sacral roots, and is interlaced with somatic, sympathetic and parasympathetic nerves. The somatic nerves conduct somatic sensory and motor signals from/to the pelvis and the lower extremities, they control the external urethral and anal sphincters, they contribute to sexual functions and conduct somatic pain. Sympathetic activity contracts the internal urethral and anal sphincters and relax other smooth muscles, while parasympathetic activity has the opposite effect. Both autonomous systems contribute to sexual activity and conduct visceral pain [5]. Pelvic trauma may injure any of these neural structures and chronic neural impairment has a great impact on quality of life after pelvic trauma [1, 2, 45, 165].

The initial neurological examination is difficult in severe pelvic trauma. Prehospital personnel may have noted presence or absence of limb movement before medication/intubation, but most severely injured patients are intubated before ER arrival. The majority of those who are not, have a reduced level of consciousness with pain and analgesics rendering neurological examination incomplete. The comatose or intubated patient may be investigated for anal sphincter tone and for presence of the bulbospongiosus (Osinski) reflex, but even these signs may be affected by pharmacological muscle relaxation. A dilated urinary bladder may result from neural damage but may also be due to other causes. An alert patient in a quiet setting should be subjected to a thorough neurological exam using i.e. the system from the American Spinal Injury Association [149]. With some intubated patients, it is desirable to reduce sedation in the ICU to better assess their neurologic status, and the use of electrophysiological investigations in the preoperative assessment has been reported [17]. Often, however, early treatment decisions must rely on radiographic findings rather than clinical signs.

Neurologic deficits may result from stretch, tear/avulsion or compression injuries [86]. In the clinical setting stretch and compression may be relieved by non-operative, or most often surgical interventions, while there are so far no available treatments for nerve tears or avulsions in the pelvic region. Neurological deficits related to sacral fractures Zone II or III [45, 68], to SI-joint dislocations and to injuries in the anterior pelvic ring may occasionally be due to nerve entrapment, but usually result from stretch or tear. Surgical treatment focuses on skeletal reduction and stabilization. Also in Zone II fractures, reduction and stabilization may relieve nerve injuries arising from stretch or compression [27], and nerve root decompression through the fracture has been reported [151].

Transverse sacral fractures in Zone III usually coexist with vertical fracture lines, constituting variations of a spinopelvic dissociation as stability between the spine and the pelvis is compromised. The transverse fracture component often narrows the spinal canal, and injury to the cauda equina has been reported in 33% of spinopelvic dissociations [117]. Lindahl reported that the degree of sagittal translation was associated with the initial degree of neurological deficit in the extension type injuries and suggested that degree of translation should be incorporated into the classification [104].

With transverse sacral fracture types, both closed and open reduction may fail to restore sagittal alignment [104, 115], still leaving a narrowed spinal canal. Clinical reports suggest that with this injury pattern sacral laminectomy may improve nerve recovery [18, 104, 157, 170, 183], and foraminal debridement through the laminectomy is also reported [94]. Neurological improvement may, however, also occur without laminectomy [4]. Concerning timing, sacral injuries compromising the cauda equina should be managed urgently, but not necessarily emergently [128]. A 2017 review of the literature concerning timing of decompression could not conclude that decompression within 72 h entailed better neurologic recovery than after 72 h, but the evidence was weak [95]. A consensus report from 2023 recommended laminectomy in the case of imaging suggestive of compression, together with complete or progressive neurological deficit [6].

In our practice, spinopelvic dissociation with neurological deficit and CT indicating a compromised spinal canal is treated with urgent, but not emergent surgery. If radiology indicates high-grade neural compression, surgery may be decided upon even if the patient is not available for neurological examination. Timing depends on the patient’s over-all condition. Laminectomy is usually performed if not contraindicated by e.g. severe soft tissue injury, and foraminal debridement is attempted if warranted by the fracture configuration.

#### Open pelvic fractures

Open pelvic fractures are rare, but constitute a subset of devastating pelvic injuries with reported mortality rates from 4% up towards 60%, more so in the older patient series [80, 112, 118, 137, 138, 145, 146]. The open pelvic fracture communicates with the outside environment, rectum or vagina, and is often associated with severe pelvic floor disruptions, with consequent loss of internal tamponade effects and profuse bleedings. Thus, acute hemorrhage and later sepsis are the major contributors to the high mortality rates [26, 41, 66, 67, 131, 162]. Improved initial algorithms for hemorrhage control have contributed significantly to later years’ improved mortality rates [73], but standard algorithms for exsanguinating pelvic trauma have not sufficiently addressed the differences and critical decision points for the open fractures [39]. Even though there is a lack of standardized management algorithms, modern evidence -based approaches encompasses a multi-disciplinary team approach involving relevant expertise like trauma surgeons, orthopedic surgeons, urologists and plastic surgeons [42, 174].

Several attempts to classify the associated soft tissue injuries in open pelvic fractures have been published, like modifications of Gustilo and Anderson’s well known classification [76] and Faringer’s three zones of injury based on wound localization [53]. Jones further included pelvic fracture instability and presence or absence of a rectal or perineal wound [92], and Cannada et al. could show that unstable fractures with a perineal or rectal wound had a mortality rate of 38% compared to an overall mortality of 23% [26].

Classically, an open pelvic fracture prompted a recommendation for colostomy to avoid soft-tissue sepsis [137]. Faringer, however, reported a 31% wound sepsis rate with colostomy and 19% without colostomy, and recommended a strictly selective approach to fecal diversion [53], also largely confirmed by later authors [181]. Thus, recent literature suggests that a diverting colostomy can reduce septic complications in open fractures with perineal and/ or rectal wounds, whereas most open pelvic fractures do best without a diverting colostomy. Classification of open pelvic fractures still remains important to the understanding of the extent and severity of the injury, as well as the need for fecal diversion, potential outcome and mortality [179].

The initial treatment of the open pelvic fracture focus on hemorrhage control, as well as meticulous wound debridement and administration of broad-spectrum antibiotics. Initial fracture stabilization can be performed with a binder or an external fixator according to wound localization and need for external access, i.e. for wound packing. Persistent hemorrhage after initial fracture reduction and stabilization prompts immediate packing of the external wounds and further hemostatic emergency procedures as previously outlined, utilizing AE, PP, and REBOA according to institutional access and protocols.

Removal of packs should be performed before 24 h. Serial debridements may be needed, and final wound closure is performed after satisfactory surgical debridement(s) according to wound size and localization. Exposed bony tissues must be handled at earliest convenience and can most often be covered with local skin or muscle flaps, even though more advanced flap surgeries may be required in select cases [58, 73]. The use of negative pressure wound therapy (NPWT) in open fracture treatment is debatable [35, 105], in our opinion vacuum-sealed dressings are very useful for wound drainage and significantly ease nursing in the ICU. However, NPWT must not be an excuse for delaying adequate surgical debridements and wound coverage.

## Conclusions

Mortality rates after bleeding high energy pelvic injuries are reduced during the last two decades due to modern DCR algorithms based on hemostatic transfusions, permissive hypotension and immediate hemorrhage control by operative or angiographic means. For the bleeding pelvic fracture in the hemodynamically unstable trauma patient, fracture reduction with binder stabilization, and subsequent AE and/or PP remain the mainstay of initial treatment. These hemostatic procedures address different bleeding sources and should be considered complementary rather than mutually exclusive. Utilizing a hybrid ER, AE may represent the main option for damage control in the bleeding pelvic trauma patient, and PP may be reserved for the patient in extremis. REBOA constitutes a valid hemostatic option in select cases, but merely represents a bridge to other invasive hemostatic procedures and its definitive role in pelvic trauma is not clearly established. Local and geographic differences in resuscitation protocols exist, however, and there is a lack of clear evidence in the literature to support one for another.

Associated nerve and genitourinary tract injuries negatively affect the long-term outcomes. A high level of suspicion is needed for early diagnosis and the initial treatment needs to follow predetermined pathways to avoid severe complications and optimize functional results. Open pelvic fractures are still hampered with high morbidity and mortality, and early treatment must focus on hemorrhage control and adequate soft tissue handling. Fecal diversion is indicated only in select cases, like rectal or peri-rectal injuries.

## Data Availability

Further case data are available on request from the corresponding author, JEM. The data are not publicly available due to their containing information that could compromise the privacy of patients.
